# The relationship between childhood maltreatment and learning engagement of high school students: the role of growth mindset and beliefs about adversity

**DOI:** 10.3389/fpsyg.2023.1222855

**Published:** 2023-09-05

**Authors:** Xintong Zhao, Lijuan Quan

**Affiliations:** School of Educational Science, Anhui Normal University, Wuhu, China

**Keywords:** high school students, childhood maltreatment, learning engagement, growth mindset, beliefs about adversity

## Abstract

**Objective:**

To explore the relationship between childhood maltreatment, growth mindset, beliefs about adversity and learning engagement among high school students.

**Methods:**

Research participants were selected by random cluster sampling.652 high school students (50.2% male and 49.8% female) from five high schools were investigated using paper-pencil survey versions of Child Trauma Questionnaire, The Utrecht Work Engagement Scale-student, Growth Mindset Scale, and The Beliefs About Adversity Scale.

**Results:**

Childhood maltreatment had a significant negative effect on high school students’ learning engagement. Childhood maltreatment directly predicted high school students’ learning engagement and also had an indirect negative predictive effect on learning engagement *via* growth mindset

**Conclusion:**

Growth mindset plays a mediating role between childhood maltreatment and learning engagement. The beliefs about adversity moderated the relationship between childhood maltreatment and growth mindset, as well as the relationship between childhood maltreatment and learning engagement. This study has empirical implications for helping high school students who have experienced childhood maltreatment to develop growth mindset and teaching students to adopt positive adversity beliefs in response to trauma during psychological interventions, thereby increasing high school students’ engagement in learning.

## 1. Introduction

Learning engagement refers to a continuous, positive emotional state that individuals hold during the completion of their studies, and is expressed in three dimensions: vitality points to resilience, dedication means full enthusiasm for learning, and concentration refers to the degree of focus (Schaufeli et al., 2002). In recent years, learning engagement has received increasing attention from researchers with the rise of positive psychology (Jung and Lee, 2018). As an important predictor of student academic achievement and dropout rates, learning engagement can visually demonstrate students’ learning effort (Liu, 2019) and it can effectively predict students’ academic performance or further education (Wang, 2022). Through a study on the learning engagement of 1,411 migrant children, Liu found that 15.77% of migrant children had low engagement in learning (Liu, 2021). In a study of the current state in learning engagement among middle school students, it was found that middle school students’ learning engagement was significantly higher than that of high school students, and from junior to senior years, middle school students’ learning engagement demonstrated a trend of decreasing with increasing grade level (Liu, 2015). At the high school level, high school students face the pressure of the college entrance examinations. With the increased difficulty of learning, a state of high commitment to learning is even more necessary. Therefore, by studying the influencing factors and mechanisms of learning engagement of high school students, we can enrich the research results in related fields and provide practical and effective help for high school students in need.

From the integrated developmental model of children’s learning engagement, it is known that both external factors (e.g., family environment, parenting style, etc.) and internal factors (e.g., cognitive ability, emotion regulation, etc.) can have an impact on learning engagement (Wang et al., 2019). Current domestic and international studies on the factors influencing learning engagement have mostly focused on the influence of recent environmental characteristics, lacking research on individuals’ own internal factors and early adverse environmental characteristics (Liu, 2021). Childhood maltreatment as an early adverse experience refers to a variety of neglect or abuse by children’s primary caregivers while growing up, which includes five dimensions: physical abuse, emotional abuse, sexual abuse, emotional neglect, and physical neglect (Bernstein et al., 1998). Some studies have shown that such early adverse experiences not only cause immediate or acute short-term harm to children, but also have long-term adverse effects on the victims’ psychological health, personality traits, and academic achievement (Yue et al., 2020). According to limited resource model of self-control, individuals have limited resources for self-control, and when individuals are exposed to traumatic and stressful events, their self-control energy will be reduced (Oaten and Cheng, 2006), resulting in a lack of ability to resist temptation, which could then have a negative impact on learning engagement (Zhu et al., 2018). Empirical studies have shown that individuals with experiences of childhood maltreatment have less self-control, prefer immediate rewards, and have difficulty showing persistence on learning to engage in such a sustained, focused activity (Simmen-Janevska et al., 2015); Zhang et al. (2023) study on middle school students found that negative parenting styles can exacerbate family conflicts, making it difficult for adolescents to fully engage in learning. Whereas childhood maltreatment, as a kind of negative parenting behavior committed by the primary caregiver, is prone to lead to the alienation of parent–child relationship and reduce their level of learning engagement (Tian et al., 2018; Peng et al., 2020). These suggest that childhood maltreatment is an important factor affecting individuals’ learning engagement, and the higher the score of childhood maltreatment, the lower the individuals’ learning engagement will be. Accordingly, hypothesis one is proposed: childhood maltreatment may significantly and negatively predict individuals’ learning engagement.

Growth mindset refers to an individual’s belief that his or her abilities can be molded and can evolve with learning (Peng and Li, 2004), it is a concept based on the theory of implicit intelligence (Song et al., 2022). According to Dweck, individuals with growth mindsets hold a “competence growth perspective,” which is characterized by growth and shaping of intelligence (Dweck, 2006; Blackwell et al., 2007). Throughout domestic and international research, it has been found that thinking patterns are more significantly culturally differentiated (Yan et al., 2021). A cross-cultural study found that cultural beliefs influence how individuals define their beliefs about thinking. Compared to students in Western cultures (e.g., “Wisdom is God-given”) who are willing to attribute success to talent, Chinese students in Eastern Confucian cultures (e.g., “Continual dropping will wear away a stone”) are more likely to attribute success to effort (Bernardo et al., 2021). According to the organism-environment interaction model, the development of growth mindset is influenced by the interaction of internal individual factors and external environmental factors (Wang, 2022). Family environment is associated with adolescents’ growth mindset. A good parent–child relationship can promote the development of growth mindset, and when the parent–child relationship plays poorly, the growth mindset will be affected (Kern et al., 2015). In addition, Thought Pattern theory suggests that mindset can help individuals construct a coherent meaning system that influences individuals’ setting of academic goals and retention of learning status (Jiang et al., 2019). In a study of 365 high school students in China, researchers found that students with high levels of growth mindset were more motivated to learn and more engaged in their studies (Liu, 2022). Growth mindset can motivate college students to seek strategies to cope with challenges and maintain an active, sustained state of learning (Hu et al., 2022). Students with growth mindset are prone to set learning-oriented goals in their studies, when they faced with failure, they will attribute it to variable factors such as efforts, invest more time or energy to continuously improve their current situation and enhance their academic achievements (Yeager and Dweck, 2020). Empirical studies have shown that the family environment, which is an important micro-environment for individual growth and development, can have an impact on the development level of individual growth mindset (Wu, 2021). More importantly, growth mindset positively predicts the desirability and engagement of individuals in learning (Liu, 2016). This suggests that childhood maltreatment can hinder the formation of individuals’ growth mindset, which in turn has an impact on their learning engagement. Thus, hypothesis two is proposed: growth mindset may play a mediating role in the relationship between childhood maltreatment and learning engagement.

The beliefs about adversity refer to an individual’s perception of the nature of adversity, including the causes, consequences and how to cope with it (Shek, 2005). In recent years, beliefs about adversity, as a value that is highly characteristic of Chinese culture, have received increasing attention from researchers for the role they play in disadvantaged children (Shek, 2005). Beliefs about adversity in different cultures can not only influence how individuals define and perceive adverse situations, but also their coping resources and coping behaviors (Shek et al., 2003). Beliefs about adversity in Chinese culture can be divided into two categories: First, positive beliefs about adversity, emphasizing that the positive value of adversity and the ability of human beings to overcome it, which are basically shaped by Confucianism. For example, “where there is a will, there is a way,” “no pain, no gain,” etc.; The second is the negative beliefs about adversity, emphasizing the insignificance of human beings in the face of adversity and the negative impacts of adversity, which are influenced by Buddhism and Taoism. For example, “poverty stifles ambition,” “good and bad situations are destined to happen” (Shek, 2004). According to organism-environment interaction model, not all children growing up with childhood maltreatment will have lower learning engagement behaviors; instead, the relationship between family risk factors (e.g., childhood maltreatment) and developmental outcomes (e.g., growth mindset, learning engagement) may be influenced by individual positive traits (e.g., beliefs about adversity). Specifically, individuals who have experienced childhood maltreatment perceive themselves to be in adversity, and beliefs about adversity facilitate high school students who have experienced childhood maltreatment to correctly perceive adversity and thus reduce the negative effects of childhood maltreatment. According to moderation model theory of adversity beliefs, the regulation mode of adversity beliefs is divided into two types: “a lamp in a darkroom” and “a drop in the bucket”: Firstly, in “a lamp in a darkroom” model, positive traits can buffer the harmful effects of risk factors, and when risk factors are high, the protective effect of positive traits is greater (Wang et al., 2017). Second, In “a drop in the bucket” model, positive traits are not sufficient to buffer the harmful effects of more risk factors, and as individuals’ risk factors increase, the protective effect of beliefs about adversity will diminish (Li et al., 2012). Empirical studies have also shown that when individuals face negative situations, beliefs about adversity can not only reduce the negative effects of risk factors, but also have a positive psychological contribution to the individual (Zhao et al., 2013). For example, In a study of 650 migrant children, Liu and Wang (2018) found that adversity beliefs can mitigate the extent of the effect that daily stress had on children’s sense of integrity. Yang and Huang (2020) studied single-parent left-behind children and found that beliefs about adversity moderated the relationship between emotional neglect and game addiction, and the moderating model was “a drop in the bucket” model. Therefore, beliefs about adversity may act as a powerful protective factor to reduce the negative effects of risk factors (e.g., childhood maltreatment) and make high school students more self-reliant and self-sufficient. Accordingly, hypothesis three is proposed: beliefs about adversity could moderate the relationship between childhood maltreatment and growth mindset, as well as childhood maltreatment and learning engagement.

**Figure 1:**
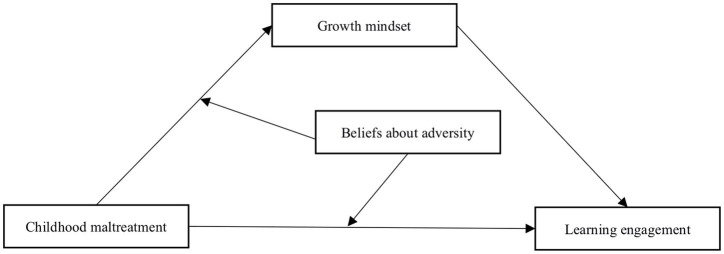
Hypothetical model diagram for the study.

A review of the literature reveals that previous studies on childhood maltreatment have focused on patients with physical and psychological disorders, and studies on non-diseased groups have focused on adults or college students, with fewer studies on high school students; in addition, studies on learning engagement have mostly been linked to teacher support, classroom climate, and self-esteem (Li, 2012; Zhang, 2012); Finally, no research has been conducted to examine the moderating mechanisms of beliefs about adversity between childhood maltreatment and growth mindset, childhood maltreatment and learning engagement. Therefore, the proposed research will discuss the mediating role of growth mindset and the moderating role of beliefs about adversity, which can enrich the research results in the field of childhood maltreatment and learning engagement to a certain extent and provide practical and effective help for high school students who have difficulties in learning engagement. A hypothetical model was generated and is shown in Figure 1.

## 2. Study design

### 2.1. Participants and data collection

Research participants were selected by random cluster sampling. Potential participants were recruited from Wuhu, Fuyang, and Huainan, China from January to March 2023. We conducted paper-pencil survey to assess childhood maltreatment, learning engagement, growth mindset, and beliefs about adversity among high school students. The survey was anonymous. Using this method, we gathered 652 high school students. Among them, 220 (33.7%) were freshman, 205 (31.4%) were sophomore and 227 (34.8%) were juniors; 327 (50.2%) were male students and 325 (49.8%) were female students; 299 (45.9%) belonged to the Social Science and 353 (54.1%) to the Natural Science; 337 (51.7%) from urban areas and 315 (48.3%) from rural areas, they effectively completed the questionnaires (Table 1). This project was approved by the Research Ethics Committee of Anhui Normal University. All participants understood the purpose of this study and provided written informed consent to participate. After the questionnaire were completed, the participants were informed that school psychologists were available to provide any psychological/counseling services if needed.

**Table 1:** Descriptive table of participant characteristics.

Variables	Categories	Number of sample	Percentage(%)
Gender	Male	327	50.2
	Female	325	49.8
Grade	Freshman	220	33.7
	Sophomore	205	31.4
	Junior	227	34.8
Household registration	Urban	337	51.7
	Rural	315	48.3
Major	Social science	299	45.9
	Natural science	353	54.1

### 2.2. Instruments

#### 2.2.1. Child trauma questionnaire

The study used the Child Trauma Questionnaire, which was compiled by Bernstein et al. (2003) and revised by Zhao et al. (2005). The questionnaire consists of 28 items, including 5 dimensions, which are emotional abuse, physical abuse, sexual abuse, emotional neglect, and physical neglect. Sample items include, “I was valued by someone in my family,” “My family was a source of strength and support for me at that time,” etc. The Likert 5-point scale was used to score from “never” to “always,” and the score is “1” to “5,” respectively. The higher the total score, the more severe the abuse during childhood. In this study, Cronbach’s alpha coefficient of the scale was 0.94.

#### 2.2.2. The Utrecht work engagement scale-student

The study used The Utrecht Work Engagement Scale-student, which was compiled by Schaufeli et al. (2002) and revised by Fang et al. (2008). It has been demonstrated that the scale is applicable to the Chinese high school student population (Zhang et al., 2008). The questionnaire consists of 17 items, including three dimensions: vitality, dedication, and concentration. Sample items include, “When I wake up in the morning, I am happy to study,” “When I study, I am energized and motivated,” etc. The Likert 7-point scale was used to score from “never” to “almost every day,” and the score is “1″ to “7,” respectively. The higher the total score, the higher the levels of learning engagement. In this study, Cronbach’s alpha coefficient of the scale was 0.93.

#### 2.2.3. Growth mindset scale

The study adopted the growth mindset scale compiled by Dweck (2006). The scale consists 6 items. Sample items include, “You can learn new things, but you cannot change your basic level of intelligence,” “You can change your intelligence to a great extent, no matter who you are,” etc. The Likert 6-point scale was used to score from “completely disagree” to “completely agree,” and the score is “1” to “6,” respectively. The higher the total score, the higher the individual’s level of growth mindset. The scale has been used several times in studies of Chinese college students, middle school students, and elementary school students, with good reliability and validity (Wu, 2021; Liu, 2022; Zhao et al., 2022). In this study, Cronbach’s alpha coefficient of the scale was 0.87.

#### 2.2.4. The beliefs about adversity scale

The study used the Chinese version of The Beliefs About Adversity Scale, which was compiled by Shek (2005). The scale consists of 9 questions. Sample items include, “Where there is a will, there is a way” “satisfied with what one has,” etc. The Likert 6-point scale was used to score from “strongly disagree” to “strongly agree,” and the score is “1″ to “6,” respectively. The scale has been used many times in Chinese children and adolescents with good reliability and validity (Shek, 2004, 2005; Zhao et al., 2013; Liu and Wang, 2018; Yu and Qin, 2020). In this study, Cronbach’s alpha coefficient of the scale was 0.84.

### 2.3. Statistical analysis

SPSS 26.0 software was used for data analysis and correlation analysis was used to describe the correlation of the variables. The Process macro for SPSS prepared by Andrew Hayes was used to test the model (Hayes, 2013).

## 3. Results

### 3.1. Common method biases test

To avoid serious common method bias in this study, Harman’s single-factor test was used for the common method biases test (Zhou, 2014). The results showed that there were nine factors with characteristic roots greater than one, and the variation rate explained by the first factor was 35.18%, which was less than the standard of 40%. Therefore, it can be considered that there is no obvious methodological bias in this study.

### 3.2. The correlation between the study variables

As can be seen from Table 1, childhood maltreatment significantly and negatively correlated with learning engagement (*r* = −0.64, *p* < 0.01), childhood maltreatment significantly and negatively correlated with growth mindset (*r* = −0.56, *p* < 0.01), childhood maltreatment significantly and negatively correlated with beliefs about adversity (*r* = −0.20, *p* < 0.01); growth mindset significantly and positively correlated with learning engagement (*r* = 0.54, *p* < 0.01), growth mindset significantly and positively correlated with beliefs about adversity (*r* = 0.37, *p* < 0.01); learning engagement significantly and positively correlated with beliefs about adversity (*r* = 0.36, *p* < 0.01).

### 3.3. Moderated mediation effect test

In order to explore the relationship between childhood maltreatment and learning engagement in high school students, the effects of demographic variables were excluded. After controlling additional variables, a simple mediation effect analysis was carried out using Model 4 in the PROCESS macro 3.3 version compiled by Hayes (2013). As shown in Table 2, childhood maltreatment had a significant negative predictive effect on learning engagement (*β* = −1.99, *t* = −21.12, *p* < 0.01), and this effect was retained (*β* = −1.55, *t* = −14.20, *p* < 0.01) when the mediation variables were included. Childhood maltreatment significantly and negatively predicted growth mindset (*β* = −1.13, *t* = −17.16, *p* < 0.01), and growth mindset significantly and positively predicted learning engagement (*β* = 0.38, *t* = 7.06, *p* < 0.01). In addition, the bootstrap 95% confidence interval of the mediating effect of growth mindset did not include 0 (see Table 3), which indicates that growth mindset mediated the association between childhood maltreatment and learning engagement. The direct effect (−1.55) and mediating effect (−0.43) accounted for 77.89 and 21.61% of the total effect (−1.99), respectively.

**Table 2:** Statistics and correlation analysis results (*N* = 652).

	*M*	SD	Childhood maltreatment	Growth mindset	Beliefs about adversity	Learning engagement
Childhood maltreatment	1.84	0.59	1			
Growth mindset	3.87	1.19	−0.56**	1		
Beliefs about adversity	4.09	0.88	−0.20**	0.37**	1	
Learning engagement	4.54	1.82	−0.64**	0.54**	0.36**	1

^**^p < 0.01.

**Table 3:** Test of Mediating Effect of growth mindset.

Regression equation (*N* = 652)		Fit index			Significance of correlation coefficient			
Result variable	Predictor variable	*R*	*R*^2^	*F*(df)	*B*	BootCI lower limit	BootCI upper limit	*t*
Learning engagement		0.65	0.42	94.16^**^				
	Gender				−0.14	−0.36	0.08	−1.21
	Grade				0.20	0.06	0.34	2.85**
	Household origin				−0.04	−0.26	0.18	−0.36
	Major				0.13	−0.10	0.37	1.13
	Childhood maltreatment				−1.99	−2.17	−1.80	−21.12**
Growth mindset		0.57	0.33	63.33**				
	Gender				0.03	−0.12	0.19	0.43
	Grade				0.20	0.10	0.29	3.94**
	Household origin				−0.07	−0.22	0.08	−0.91**
	Major				−0.02	−0.18	0.15	−0.22
	Childhood maltreatment				−1.13	−1.26	−1.00	−17.16**
Learning engagement		0.68	0.46	92.69**				
	Gender				−0.15	−0.36	0.06	−1.38
	Grade				0.13	−0.01	0.26	1.84
	Household origin				−0.01	−0.22	0.20	−0.12
	Major				0.14	−0.08	0.37	1.23
	Growth mindset				0.38	0.28	0.49	7.06**
	Childhood maltreatment				−1.55	−1.77	−1.34	−14.20**

^**^p < 0.01.

**Figure 2:**
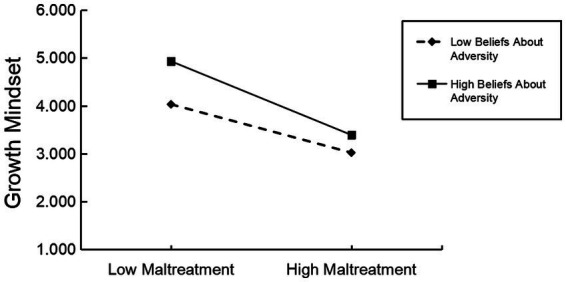
Moderating role of beliefs about adversity on childhood maltreatment and growth mindset.

**Figure 3:**
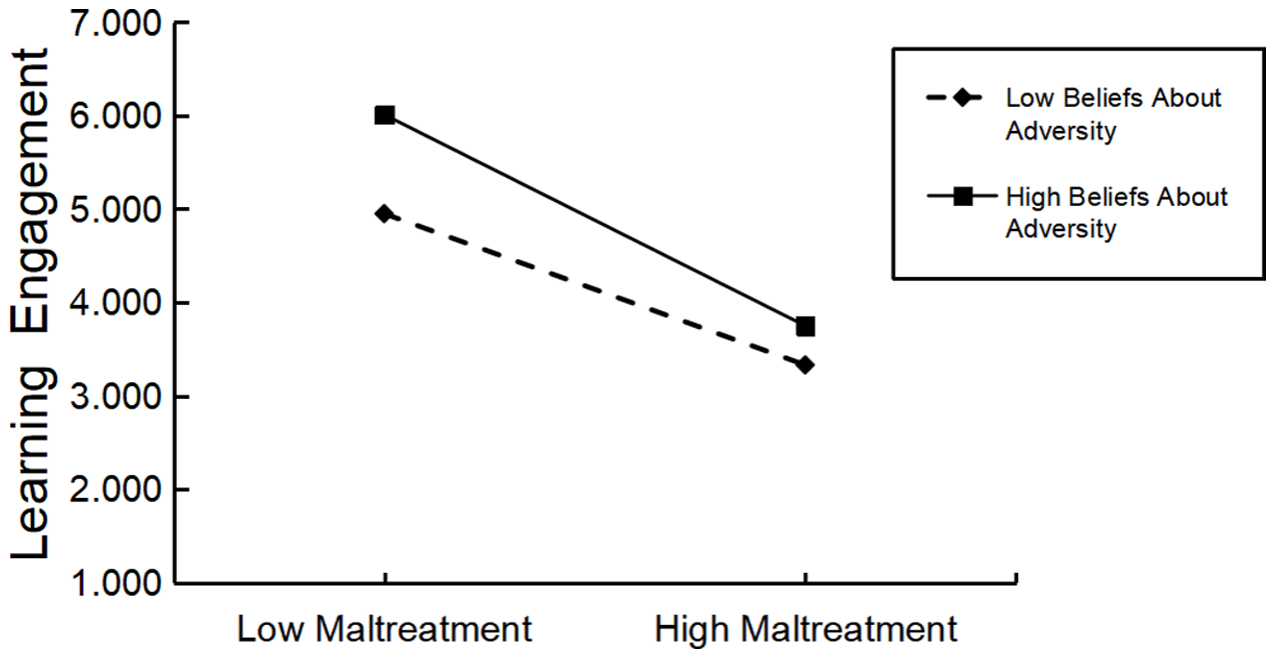
Moderating role of beliefs about adversity on childhood maltreatment and learning engagement.

The PROCESS macro Model 8 compiled by Hayes was used to test the moderated mediation effect (Fang and Wen, 2018). The results are shown in Table 4. When beliefs about adversity was added to the model, which would moderate the direct effect of childhood maltreatment on learning engagement, and also moderate the indirect effect of childhood maltreatment on growth mindset (*β* = −0.31, *t* = −3.55, *p* < 0.01; *β* = −0.26, *t* = −4.25, *p* < 0.01), indicating that beliefs about adversity not only the direct effect of childhood maltreatment on learning engagement, but also the indirect effect of childhood maltreatment on learning engagement. To clarify the nature of the interaction terms, we used a simple slope test to analyze the moderating effect of beliefs about adversity (see Figures 2, 3).

**Table 4:** Analysis of all effects.

	Effect size	Boot SE	*t*	BootCI lower limit	BootCI upper limit	Relative effect value
Total effect	−1.99	0.09	−21.12	−2.17	−1.80	
Direct effect	−1.55	0.11	−14.20	−1.77	−1.34	77.89%
Mediating effect of growth mindset	−0.43	0.05		−0.56	−0.33	21.61%

Generally, when the level of beliefs about adversity was low (M-1SD), there was a significant negative predictive effect of childhood maltreatment on growth mindset (*simple slope* = −0.86, *t* = −11.38, *p* < 0.001); when the level of beliefs about adversity was high (M + 1SD), there was a negative predictive effect of childhood maltreatment on growth mindset predictive effect，and the effect was greater (*simple slope* = −1.31, *t* = −14.22, *p* < 0.001). This indicates that the predictive effect of childhood maltreatment on growth mindset differs at different levels of beliefs about adversity, and the higher the beliefs about adversity, the stronger the predictive relationship between the two.

As can be seen from Figure 3, when the level of beliefs about adversity is low (M-1SD), childhood maltreatment has a significant negative predictive effect on learning engagement (*simple slope* = −1.38, *t* = −11.89, *p* < 0.001); while for subjects with high beliefs about adversity (M + 1 SD), childhood maltreatment has a negative predictive effect on learning engagement, and the effect was greater (*simple slope* = −1.92, *t* = −12.91, *p* < 0.001), indicating that the predictive effect of childhood maltreatment on learning engagement differed at different levels of beliefs about adversity, and the higher the beliefs about adversity, the stronger the predictive relationship between the two.

## 4. Discussion

### 4.1. The effect of childhood maltreatment on learning engagement

The results of the study indicated that childhood maltreatment was significantly and negatively related to high school students’ learning engagement, and childhood maltreatment is able to significantly and negatively predict high school students’ learning engagement. The findings are consistent with Hypothesis 1, indicating that high school students with low childhood maltreatment are more willing to invest more energy in learning and develop a stronger interest in learning. This is consistent with the findings of Gao et al. (2020) that stress and negative life events are important risk factors that contribute to decreased learning engagement. Since ancient times, people have been adhering to Confucian ideas such as “if a son is uneducated, his dad is to blame” and “dutiful son under the sticks,” holding that obeying one’s parents means doing one’s filial duty. When children do not do what their parents expect of them, parents may take harsh disciplinary measures, resulting in emotionally and physically abusive behaviors toward their children, which may affect the mental health of the individual to a certain extent. First, experiences of childhood maltreatment as an early adverse environment can cause acute short-term harm to children (Rees, 2011), and the accumulation of short-term effects often leads to long-term effects (Bushman and Huesmann, 2006). Children with high levels of childhood maltreatment suffer from the long-term effects of childhood maltreatment through their high school years. According to helplessness theory, individuals who suffer from chronic childhood maltreatment perceive themselves as worthless and attribute negative events to their own shortcomings, which increases the individual’s susceptibility to depression (Rose and Abramson, 1992), causing psychological distress to themselves and affecting their own learning and lives. Second, according to the limited resource model of self-Control, when individuals are exposed to stressful events or situations in their lives, they need to consume their own attention to cope with them, which will consume a lot of self-control resources in the process (Huang et al., 2021). When self-control resources are insufficient, individuals are easily attracted to various temptations during learning activities, resulting in various impairment problems (Gailliot and Baumeister, 2007). In addition, while affecting frontal lobe development, childhood maltreatment can also lead to pathological changes in individuals’ HPA, causing malfunctions in their executive functions (Liu et al., 2015) and making them vulnerable to interference from other factors in learning tasks, thus affecting their own learning engagement (Ji and Wang, 2017). It can be seen that high school students with low childhood maltreatment are more likely to mobilize their attentional and cognitive resources during learning, more likely to have the ability to resist temptations, and will be more engaged in learning.

### 4.2. The mediating role of growth mindset

Growth mindset partially mediates the mechanism of childhood mistreatment’s effect on learning engagement. Specifically, childhood maltreatment can have a significant negative predictive effect on learning engagement both directly and through growth mindset, a result that is consistent with research hypothesis 2. First, according to the organism-environment interaction model, as a behavior committed by the primary caregiver, childhood maltreatment is an inseparable family environmental factor in individual growth, which will have an indelible impact on the development of individual growth mindset. Growth mindset positively predicts learning engagement, which is consistent with Liu’s findings on 352 high school students in China (Liu, 2022), contrary to the results of the Western study by Cavanagh et al. (2018). Growth mindset was not significantly associated with students’ engagement in learning, whereas students’ trust in their teachers was significantly positively associated with their learning engagement, as opposed to Chinese students’ growth mindset, which was associated with increased engagement in learning. The reason for the difference may be attributed to the cultural differences between China and the West. In China, under the traditional beliefs of “dripping water penetrates the stone” as well as the pressure of the college entrance examinations due to China’s large population base, Chinese high school students put more effort into improving their learning abilities; whereas, in the relatively free classroom environment of the West, students’ trust in their teachers and the teachers’ role in the learning environment are the key factors influencing students’ learning engagement. Second, Thought Pattern theory suggests that thinking patterns can help individuals construct coherent meaning systems and influence their goal settings, attribution styles, and effort beliefs (Diao et al., 2020). Interventions on thinking patterns can stimulate students’ internal motivation to learn, thus stimulating learning potential (Aguilar et al., 2014). For high school students under the current stage of China’s college entrance examination system, learning engagement determines academic achievement during high school, affecting high school students’ future academic development and relating to their future survivability. High school students with a high-level growth mindset will be willing to spend time and energy on their studies, and when their grades are unsatisfactory, they will actively look for reasons for their failures, willing to meet challenges, and believe that their basic competencies can be cultivated through hard work (Zhao et al., 2022).

### 4.3. The moderating effect of beliefs about adversity

Based on the verification of the existence of a mediating effect of growth mindset, this study further explored the moderating role of beliefs about adversity in this mediating path. The results found that beliefs about adversity had a moderating effect not only in the first half of this mediating path, but also in the path of the independent and dependent variables, which is consistent with research hypothesis 3 (Table 5).

**Table 5:** Moderated mediation effect test.

Regression equation (*N* = 1,136)		Fit index			Significance of correlation coefficient	
Result variable	Predictor variable	*R*	*R^2^*	*F*	*B*	*t*
Learning engagement		0.71	0.5	80.89^**^		
	Gender				−0.09	−0.88
	Grade				0.09	1.33
	Household registration				0.02	0.24
	Major				0.11	1.02
	Childhood Maltreatment				−1.65	−15.10^**^
	Beliefs about adversity				0.42	6.62^**^
	Childhood Maltreatment×Beliefs about adversity				−0.31	−3.55^**^
Growth Mindset		0.64	0.41	63.68^**^		
	Gender				0.08	1.05
	Grade				0.14	2.97**
	Household registration				−0.03	−0.41
	Major				−0.04	−0.51
	Childhood Maltreatment				−1.08	−16.70^**^
	Beliefs about adversity				0.36	8.51**
	Childhood Maltreatment × Beliefs about adversity				−0.26	−4.25**

^**^p < 0.01.

First, according to the pressure buffer model, beliefs about adversity play an important role in buffering negative aspects of stress (Cohen and Will, 1985). Based on this model, individuals’ beliefs about adversity can motivate individuals in adversity to enrich their experiences and make appropriate coping strategies, thus promoting individual mental and action development and proactive coping in adversity (Shek, 2005; Tian et al., 2012; Zhao et al., 2013). The present study again supports this finding, showing that beliefs about adversity moderate the negative prediction of growth mindset by childhood maltreatment, as shown by the fact that childhood maltreatment significantly and negatively predicted individuals’ growth mindset in both high and low adversity beliefs conditions, but the predictive power of childhood maltreatment on growth mindset was higher in high adversity belief conditions. As a new psychological resource, beliefs about adversity can enhance individuals’ self-efficacy and motivate them to be more willing to face reality and try to solve problems (Bao et al., 2014). Notably, the findings are consistent with “a drop in the bucket” model, meaning that the protective effect of beliefs about adversity are less pronounced in individuals with higher levels of childhood maltreatment. The results of this study are consistent with those of Yang and Huang (2020), when individuals are placed in risky factors, their development remains influenced by risky factors once the power of the risky factors is too strong and exceeds the individual’s psychological capacity (Chen et al., 2017).

Second, the findings showed that beliefs about adversity moderated the negative prediction of childhood maltreatment on learning engagement. Further analyses revealed that the protective effect of beliefs about adversity was more pronounced with low childhood maltreatment. Individuals with high childhood maltreatment had lower levels of learning engagement relative to those with low childhood maltreatment, and the protective effect of beliefs about adversity on learning engagement decreased as the level of childhood maltreatment increased. There are two possible reasons for these results: First, according to the stress vulnerability hypothesis, individuals with positive traits lose their buffering effect in high stress situations. Individuals with positive qualities develop well in low-stress environments, but deteriorate rapidly in high-stress environments (Vanderbilt-Adriance and Shaw, 2008). When individuals experience increasing levels of childhood maltreatment, the buffering effect of protective factors against negative factors can be reduced (Ye et al., 2014). High school students’ physical and mental development is still immature, and when faced with more stressful life events, the role of adversity beliefs in promoting learning engagement and growth mindset is diminished; secondly, the negative effects of childhood maltreatment and the protective role of beliefs about adversity are opposed to each other, and the process of confrontation between the two may cause psychological distress to high school students in adolescence and interfere with the release of their positive psychological resources (Vanderbilt-Adriance and Shaw, 2008).

Finally, for a high school student who holds the positive beliefs about adversity that are promoted by Chinese culture, “Only by enduring hardship can one become a superior person” may become their usual credo. However, in the context of high levels of trauma and stress, such beliefs may be gradually eroded or altered, resulting in beliefs that do not correspond to the objective reality. In this way, the individual’s positive adversity beliefs may become a kind of stress, which is not protective or less protective; while for a high school student who holds negative adversity beliefs promoted by Chinese culture, “Poverty stifles ambition,” etc., may become their usual creed, and they may be reluctant to take appropriate measures to cope with traumatic or stressful events, which may result in the individual’s reluctance to take appropriate measures to cope with traumatic or stressful events. When they encounter traumatic and stressful events, they will be reluctant to take appropriate measures to cope with them, thus, the adversity beliefs held by individuals will not be utilized, and will not have a protective effect.

## 5. Research significance and shortcomings

This study conducted a investigation into the mechanism of the relationship between childhood maltreatment and learning engagement of high school students, verified the mediating role of growth mindset and the moderating role of beliefs about adversity, and provided new ideas for basic educators to improve the learning engagement of high school students. In terms of theory, this study found that childhood maltreatment can affect learning engagement through the mediating role of growth mindset, and beliefs about adversity moderate the relationship between childhood maltreatment and learning engagement, as well as between childhood maltreatment and growth mindset. This study enriches the research findings in this area and deepens the understanding of the relationship between childhood maltreatment and learning engagement in high school students. In terms of practice, this study provides some implications for improving the engagement of high school students in learning. First, reduce the likelihood of childhood maltreatment, lessen the incidence of trauma, mobilize the full range of protective resources around children who have experienced childhood maltreatment, and alleviate the adverse emotions. It is known from Basic Needs theory that when an individual’s basic psychological needs are met, the individual’s internal motivation will also be enhanced and the level of learning engagement will increase. Thus, parents need to provide a benign environment for children in the process of their growth or development and pay attention to their psychological development; teachers can guide children to conduct positive thinking training, consider traumatic memories only as events in the mind, and cultivate positive emotions in children. Secondly, teachers can conduct lectures or courses related to growth mindset in the education process, dare students to explore themselves in challenging learning tasks, motivate students to make reasonable attributions for failures, and cultivate growth mindset in students so as to increase their level of learning engagement. Finally, it is worth noting that although higher beliefs about adversity mitigate the negative effects of childhood maltreatment on growth mindset and childhood maltreatment on learning engagement, the results of this study also suggest that beliefs about adversity are more conducive to the development of high levels of growth mindset and high levels of learning engagement in individuals with low levels of childhood maltreatment relative to high levels of childhood maltreatment. This result again reminds us that the protective effect of beliefs about adversity should not be overstated, and that the protective effect of beliefs about adversity will be weakened when the negative factors exceed the individual’s ability to cope with them. Therefore, in the actual education work, in order to promote high school students’ growth mindset and learning engagement, we should start from both trauma and protective factors: not only should we reasonably use the traditional Chinese culture of “adversity and transcendence” to help high school students understand adversity and form positive adversity thinking, but we should also regulate the source of trauma, so that all kinds of factors that promote adversity can be used. We should not only help high school students understand adversity and form positive adversity thinking, but also regulate the source of trauma, so that all kinds of facilitating factors can work stably.

This study has certain shortcomings that need to be further improved in the follow-up study, specifically in the following aspects: first, this study is a cross-sectional study, it is not easy to clarify the causal relationship between variables, future experimental studies or follow-up studies can be used to test the mediating and moderating effects in this study; Second, the study used questionnaires to examine the impact of childhood maltreatment on high school students’ learning engagement. It is difficult to avoid the words “abuse” and “neglect” in the Childhood Trauma Questionnaire, which may lead to untrue responses from the study participants in pursuit of social approval, and the findings may be biased by social expectations, which can be designed by experimental studies in future studies; Third, this study only selected high school students from several high schools for the survey, the number of subjects was limited, and the applicability of the study to high school students in other regions needs to be further tested. In future studies, it can be considered to administer the test in different cities or regions to expand the sample of subjects and make the study findings more applicable.

## Data availability statement

The raw data supporting the conclusions of this article will be made available by the authors, without undue reservation.

## Ethics statement

The studies involving humans were approved by the Research Ethics Committee of Anhui Normal University. The studies were conducted in accordance with the local legislation and institutional requirements. The participants provided their written informed consent to participate in this study.

## Author contributions

XZ and LQ: conceptualization, project administration, date curation, investigation, methodology, and writing – original draft, and writing – review and editing. XZ: resources. All authors have read and agreed to the published version of the manuscript.

## Conflict of interest

The authors declare that the research was conducted in the absence of any commercial or financial relationships that could be construed as a potential conflict of interest.

## Publisher’s note

All claims expressed in this article are solely those of the authors and do not necessarily represent those of their affiliated organizations, or those of the publisher, the editors and the reviewers. Any product that may be evaluated in this article, or claim that may be made by its manufacturer, is not guaranteed or endorsed by the publisher.
